# A Rare Case of Small Cell Carcinoma of the Urinary Bladder

**DOI:** 10.7759/cureus.8609

**Published:** 2020-06-13

**Authors:** Samia Hossain, Vinay Edlukudige Keshava, Arun Minupuri, Rajesh Thirumaran, Eugene Choi

**Affiliations:** 1 Internal Medicine, Mercy Catholic Medical Center, Darby, USA; 2 Hematology/Oncology, Mercy Catholic Medical Center, Darby, USA

**Keywords:** small cell cancer, bladder, turbt, chemotherapy, cystectomy

## Abstract

Small cell carcinoma of the bladder is a rare type of bladder malignancy. Based on most of the existing studies, there is an observed male predominance, usually in their sixties or seventies, and they are more likely to have a history of smoking. Additionally, there is a higher predilection for Caucasians (versus non-Caucasians). The most common presenting complaint is painless macroscopic hematuria. However, other presenting symptoms also include dysuria, difficulty voiding, weight loss, abdominal pain, nocturia, and urinary frequency. It is not uncommon to have a history of frequent urinary tract infections, ureteral obstruction, and paraneoplastic syndromes. Cystoscopy is the "gold standard" for evaluation of urinary tract lining, especially in conjunction with narrow-band imaging and biopsy. Transurethral resection of the bladder tumor (TURBT) is the next step in diagnosis and treatment that allows to precisely evaluate pathology and the extent of bladder wall involvement, and is a sufficient surgical approach for the treatment of non-muscle invasive tumors. Once tumor pathology is confirmed, a treatment plan is determined based on the staging. Although both lung and bladder small cell carcinoma have similarities in pathogenesis, genomic alterations in small cell carcinoma of the bladder are more similar to that of urothelial cancer rather than small cell lung cancer. As this is a rare subtype and only a few reported cases are available, no standard treatment regimen has been established. In localized disease, neo-adjuvant platinum-based chemotherapy with cystectomy has been shown to provide the best result in retrospective studies. As this type of cancer has a poor prognosis, in metastatic disease, palliative chemotherapy is offered. Here we present one such case of small cell carcinoma of the bladder and review the current literature.

## Introduction

Small cell carcinoma of the bladder is a neuroendocrine epithelial tumor that comprises less than 1% of bladder cancers. The most common presenting complaint is painless macroscopic hematuria. However, other presenting symptoms also include dysuria, difficulty voiding, weight loss, abdominal pain, nocturia, urinary retention due to clot formation within the bladder, and urinary frequency [1]. It is not uncommon to have a history of frequent urinary tract infections, ureteral obstruction, and paraneoplastic syndromes. Cystoscopy is the primary diagnostic modality, during which transurethral resection of the bladder tumor (TURBT) or bladder biopsy can be performed. Once tumor pathology is confirmed, a treatment plan is determined based on the presence or absence of metastases. As this is a rare subtype and only a few reported cases are available, no standard treatment regimen has been established. Based on retrospective studies, in localized disease, neoadjuvant platinum-based chemotherapy with cystectomy provides the best result. As this type of cancer has a poor prognosis, when there is confirmed metastasis, chemotherapy alone is provided for palliative treatment [2]. Due to the rare nature of this disease, reporting such cases will further our understanding of the therapeutic options and prognosis.

## Case presentation

**Figure 1:**
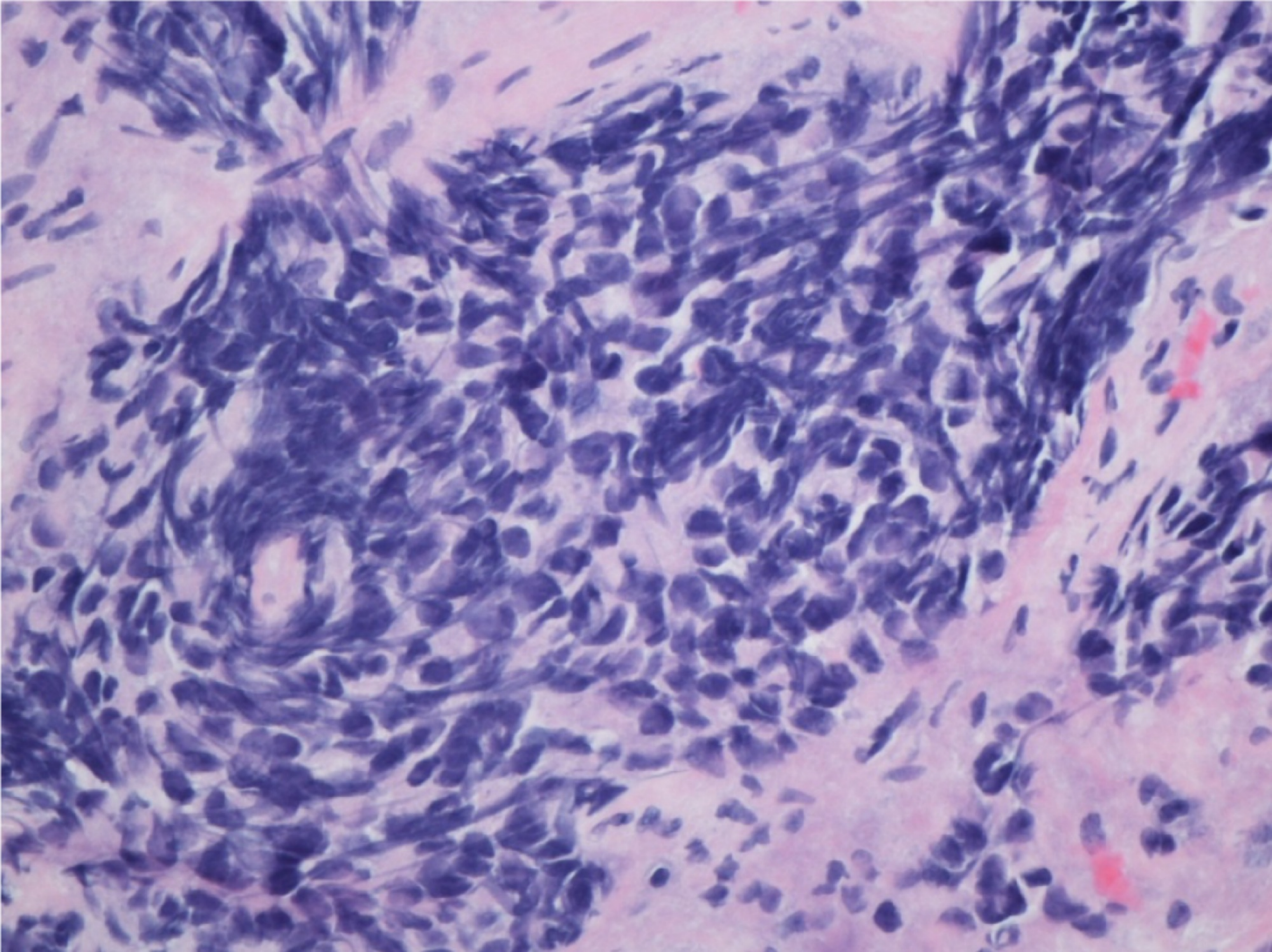
Small cells with nuclear moulding and indistinct nucleoli (H&E) at ×400 magnification.

**Figure 2:**
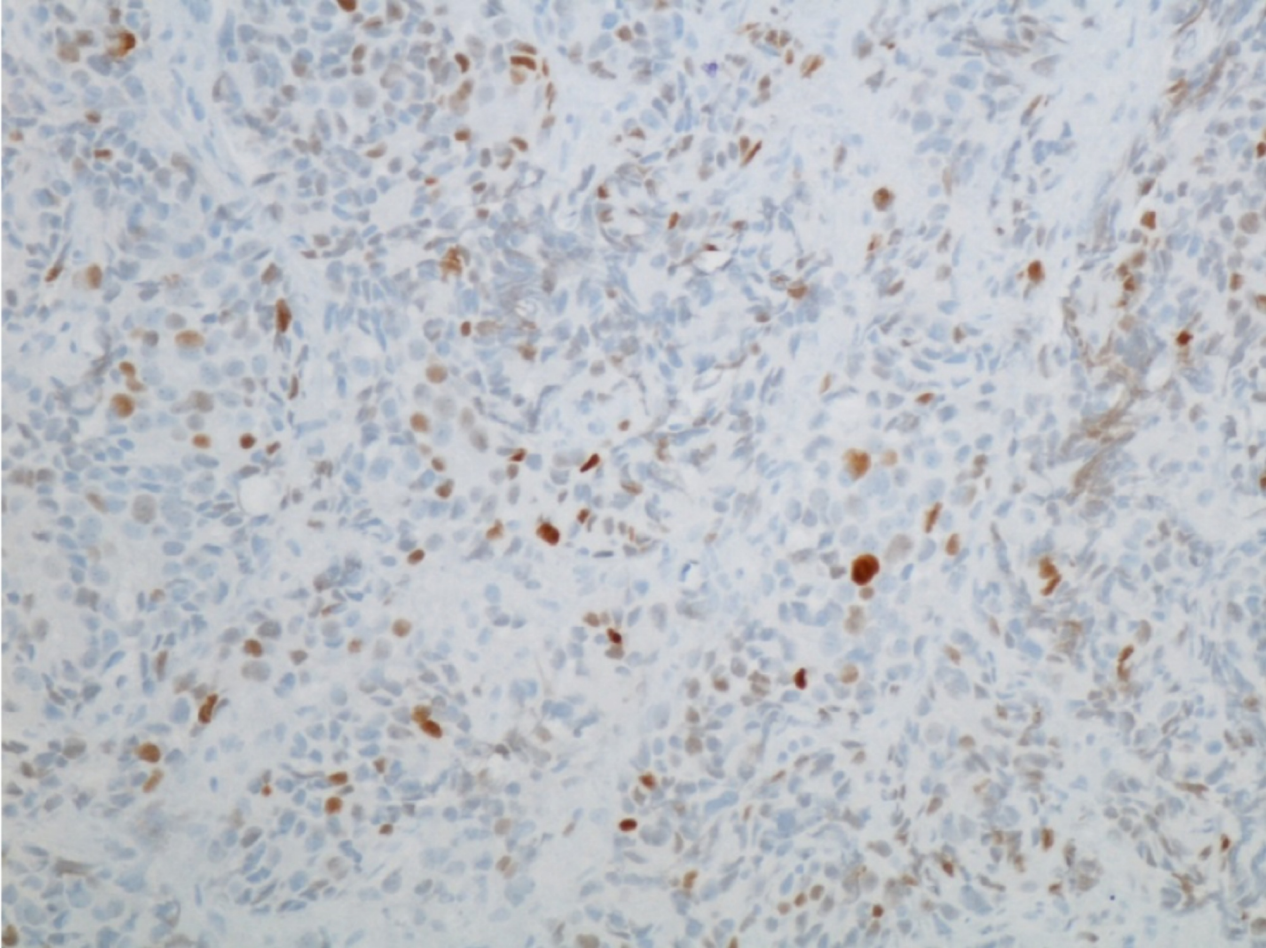
Tumor cells positive for TTF1 at ×200 magnification. TTF1, thyroid transcription factor 1

**Figure 3:**
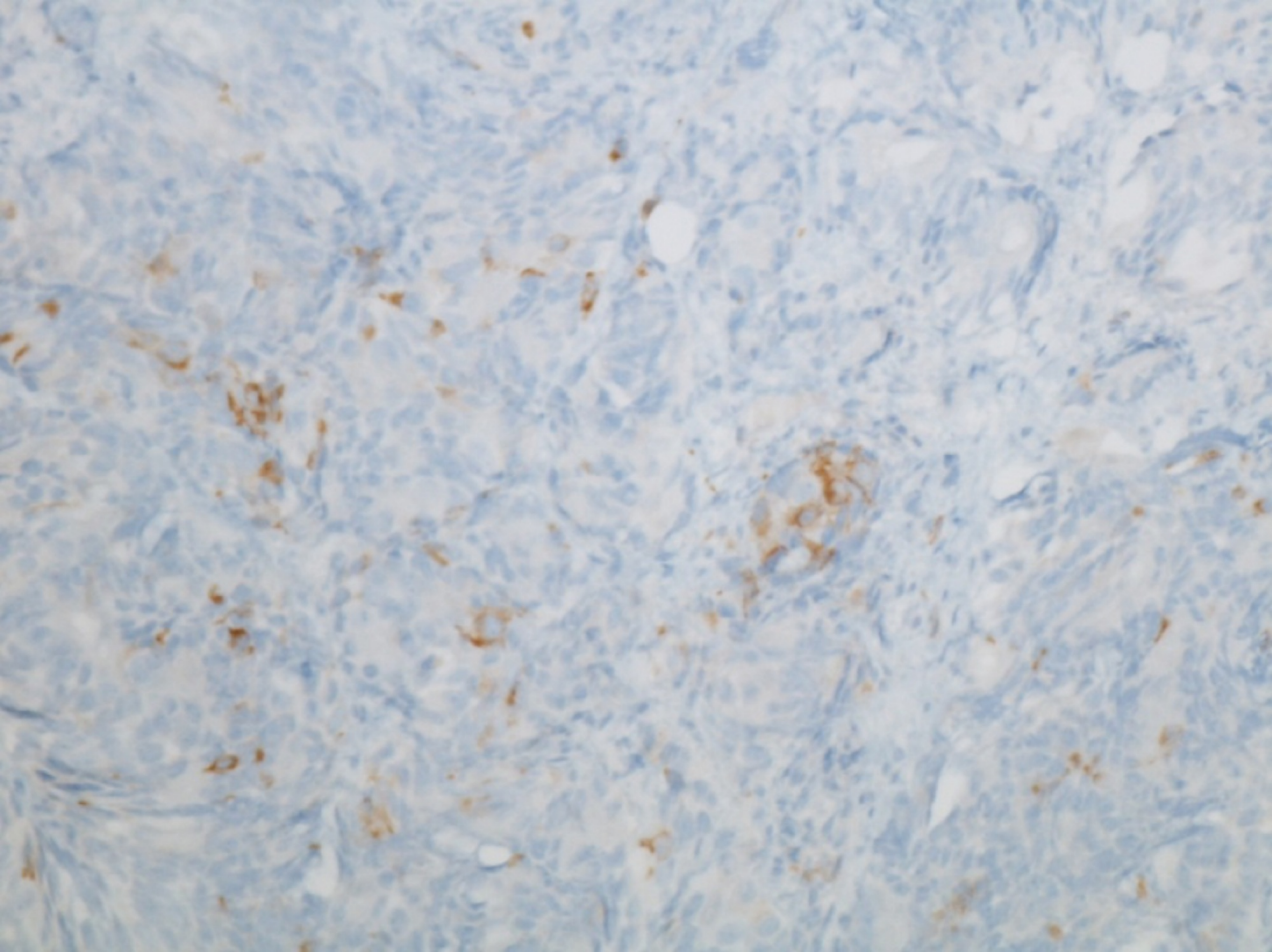
Tumor cells positive for synaptophysin (focal) at 200x magnification.

A 76-year-old man with a past medical history of chronic obstructive pulmonary disease (with history of second-hand smoke exposure and ex-cigar smoker), congestive heart failure, coronary artery disease, tachycardia-bradycardia syndrome following pacemaker placement, chronic kidney disease, atrial fibrillation (on rivaroxaban), right-sided nephrectomy (likely for renal cell carcinoma, however, the patient was unable to confirm it), hypertension, iron-deficiency anemia, and diabetes mellitus presented to our hospital for progressive worsening of shortness of breath. The patient had been admitted for monitoring and medical management of acute congestive heart failure exacerbation. Urine dipstick obtained at the time of admission showed large blood in the urine. A urinalysis, renal ultrasound, and cystoscopy were obtained for further evaluation of the hematuria, and urology service was consulted. Urinalysis showed microscopic hematuria, leukocyte esterase, white blood cells, and bacteria. Urine culture showed growth of gram-negative bacilli, and the patient was started on ceftriaxone for urinary tract infection. Histological evaluation of the urine specimen showed few atypical single and clusters of small cells present in the background with marked acute inflammation, consistent with small cell carcinoma. Renal and bladder ultrasound revealed absent right kidney and new mild left hydronephrosis, mild thickening of the bladder wall, and a small amount of debris in the bladder. Cystoscopy revealed moderate enlargement of the prostate, a significant amount of bleeding in the bladder, inability to locate the left ureteral orifice, and a significant amount of mucosal irregularity at the base of the bladder. Histologic evaluation of biopsy specimen obtained from right bladder base revealed small cell carcinoma: immunohistochemical stains were performed on block A1 and showed tumor cells positive for thyroid transcription factor 1 (TTF1), chromogranin (focal), and synaptophysin (focal), while CK7, CK20, CD56, and GATA3 were negative (Figures 1-3).

**Figure 4:**
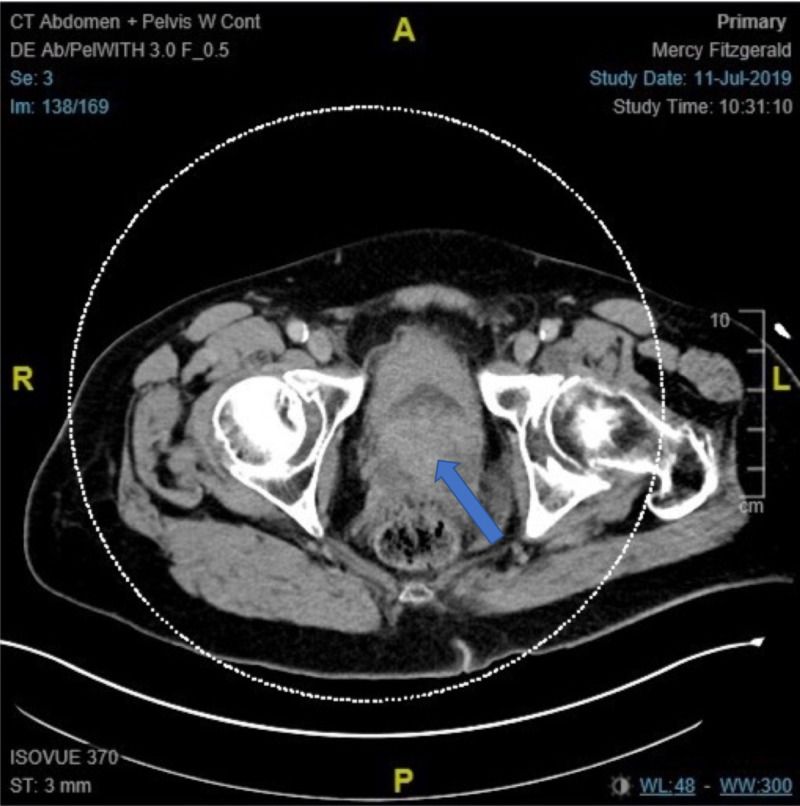
Computed tomography scan of abdomen and pelvis showing tumor of urinary bladder with involvement of prostate gland (arrow).

Computed tomography (CT) revealed an enlarged irregular appearing prostate gland protruding into the bladder base (site of biopsy-proven small cell carcinoma), bladder outlet obstruction with circumferential thickening of the urinary bladder wall, and a questionable invasion of the anterior wall of the rectum (Figure 4). 

**Figure 5:**
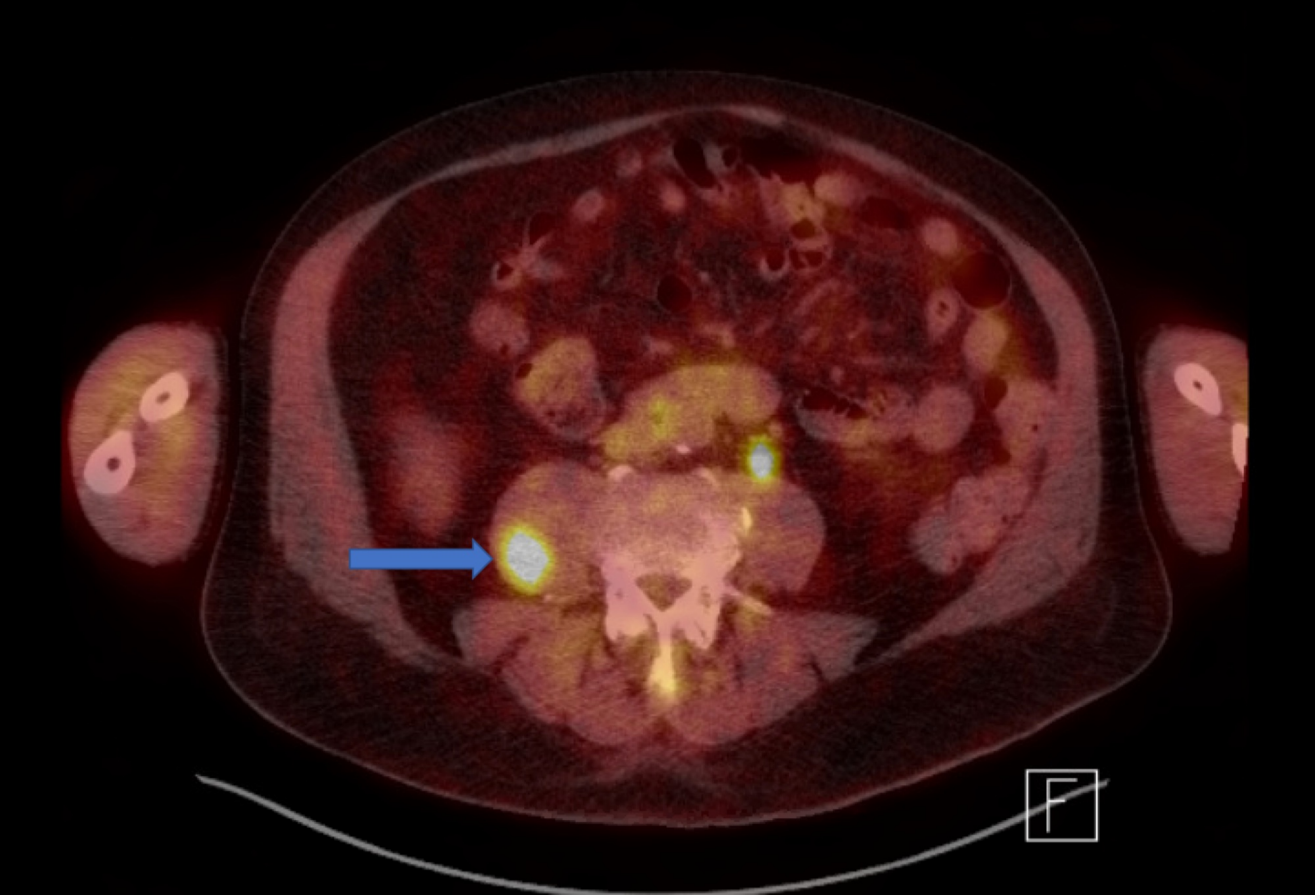
Positron emission tomography-computed tomography (PET-CT) demonstrating a 1.5-cm focus of FDG uptake in the right psoas muscle with an SUV max of 9.1 compatible with metastatic disease (arrow). FDG, F-fluorodeoxyglucose; SUV, standard uptake value.

CT imaging also revealed tumor invasion of the left ureterovesical junction with resultant left hydroureteronephrosis, in addition to lytic lesions (likely metastatic foci) in L3 and right iliac bone. On positron emission tomography-CT (PET-CT), biopsy-proven bladder mass was not visualized and intensely F-fluorodeoxyglucose (FDG) avid urine was visualized in the urinary bladder. A 1.5-cm focus of FDG uptake noted in the right psoas muscle with a standard uptake value (SUV) max of 9.1 compatible with metastatic disease (Figure 5). Lytic lesions in L3 and right iliac bone were non-FDG avid, and therefore likely to be benign lesions. Additionally, there was non-specific focal FDG uptake in the right nasopharyngeal area without corresponding mass on CT scan. The patient underwent placement of port-a-cath and was discharged with a Foley catheter for urinary retention. Two months following initial diagnosis, the patient was started on a chemotherapy and immunotherapy regimen, which included atezolizumab, etoposide, and carboplatin. Repeat cystoscopy performed four months following initial diagnosis revealed no lesions or masses. 

## Discussion

Small cell carcinoma of the bladder is an extremely aggressive neuroendocrine tumor [3]. The above case was initially suggestive of urothelial cancer, in addition to the already established diagnoses of urinary tract infection and benign prostatic hyperplasia, as the patient had initially presented with microscopic hematuria. Gross hematuria is the commonly reported initial symptom in small cell carcinoma of the bladder. Other presenting symptoms include dysuria and urinary frequency [4].

Although both lung and bladder small cell carcinoma have similarities in pathogenesis, it has been observed that genomic alterations in small cell carcinoma of the bladder resemble more that of urothelial cancer than small cell lung cancer. Therefore, most mutations contribute to carcinogenesis in an organ-specific manner rather than a cell type-specific manner [3]. Additionally, most small cell carcinomas of the bladder are usually mixed with urothelial cancer and other histologic types like urothelial carcinoma in situ (UCIS), adenocarcinoma, sarcomatous, micropapillary, squamous, and plasmacytoid, further emphasizing the idea that they may arise from a common clonal origin. Most small cell carcinomas of the bladder express neuroendocrine markers, such as chromogranin, synaptophysin, neuron-specific enolase (NSE), and CD56 [4,5]. 

Histological features include sheets and large nests of malignant cells with round to oval nuclei, which have finely stippled chromatin, abundant mitotic figures, and inconspicuous nucleoli. There is scant cytoplasm, resulting in a high nuclear to cytoplasmic ratio. These features are similar to those of small cell carcinoma of the lung. The nuclei show coagulative necrosis and oftentimes, nuclear molding as well [4].

In 90% of cases, on immunohistochemical analysis of the tumor, there was observed lack of retinoblastoma 1 (RB1) expression, which is thought to be an important genomic alteration in the development of small cell carcinoma of the bladder [4]. RB1 gene encodes a protein that acts as a tumor suppressor by regulating the cell cycle, senescence, and apoptosis. Early lesions in RB1 and TP53 have been detected in both small cell carcinomas of the bladder and lung. In small cell carcinoma of the bladder, frequency of allelic loss was 47% at TP53, the gene that holds the tumor suppressor p53 locus [5].

No standard treatment regimen has been established for small cell carcinoma of the bladder, as this is a rare histological variant and comprises less than 1% of all bladder cancers. It has been observed that treatment with neoadjuvant chemotherapy resulted in pathologic downstaging and ultimately better long-term survival, especially when followed by radical cystectomy [2]. However, once cancer metastasizes, the patient is no longer a candidate for cystectomy, and finally, the prognosis is significantly worse than in localized disease. Unfortunately, small cell carcinoma of the bladder is usually diagnosed in an advanced stage with associated widespread invasion and metastases. 

## Conclusions

Further workup for hematuria is essential to rule out any bladder malignancy, including the rare variant that is small cell carcinoma of the bladder, which has a relatively poor prognosis in advanced metastasis stage. Our patient in the aforementioned case fit several criteria for the typical demographic for small cell cancer of the bladder, including a history of smoking, being in the seventh decade of life and being of the male gender. A higher index of suspicion with further workup is warranted when encountering such patients presenting with hematuria. A better understanding of the genomic alterations that are implicated in small cell carcinoma can ideally further guide the development of more sophisticated therapeutic strategies in the future.
